# Is it feasible to perform microbiota analysis without matching antibiotic usage?

**DOI:** 10.1186/s13054-023-04435-4

**Published:** 2023-04-18

**Authors:** Mingqiang Wang, Huijun Feng

**Affiliations:** 1 Zhengzhou University People’s Hospital, Henan University People’s Hospital, Zhengzhou, China; 2 Department of Critical Care Medicine, Henan Key Laboratory for Critical Care Medicine, Zhengzhou Key Laboratory for Critical Care Medicine, Henan Provincial People’s Hospital, Zhengzhou, China; 3 Department of Neurology, The First People’s Hospital of Pinghu, Pinghu, China

**Dear Editor,**

We read the article by Sun et al. published in Critical Care with great interest [1]. The authors conducted a retrospective cohort study to analyze the correlation between gut microbiota, metabolome, and clinical outcomes in patients with sepsis. However, it should be noted that this study may suffer from serious biases and errors.

As is well known, antibiotics, especially broad-spectrum antibiotics, can cause significant disruption to the gut microbiota, including a 10,000-fold decrease in bacterial load and a decrease in diversity [2, 3]. Different antibiotic treatments can lead to the depletion of different gut microbial populations, which is a classic modeling scheme for the difficile animal model. In this study, the authors included almost all sepsis patients who had received different antibiotic treatments before sample collection, including broad-spectrum carbapenem antibiotics. However, they did not seek to include ordinary ward patients who had received carbapenem antibiotic treatment as a control group. Instead, they used healthy individuals who had not received antibiotic treatment as controls. In fact, the impact of factors such as the use of broad-spectrum antibiotics, including carbapenem antibiotics, may have a greater impact on the gut microbiota than sepsis itself. Therefore, such confounding factors make the results of this study unreliable.

Furthermore, there are also flaws in the analysis methods used in this study. Firstly, the authors used outdated OTUs for gut microbiota analysis, and ASV analysis was not performed, which weakened the power of this study. Secondly, the authors only used Lefse for differential analysis of gut microbiota between the two groups, without using more robust methods such as DESeq2. Thirdly, in this study, only a small proportion of the analysis results were adjusted for p-values, which can lead to many false positive results.

Overall, this study has value of publication, but many issues still need to be addressed.

## Data Availability

None.
